# Looking back at Covid-19 government restrictions: were local lockdown regions with tighter restrictions less adherent before the restrictions and more adherent after?

**DOI:** 10.1093/tbm/ibae061

**Published:** 2024-11-09

**Authors:** Chantal den Daas, Marie Johnston, Gill Hubbard, Diane Dixon

**Affiliations:** Health Psychology group, Institute of Applied Health Sciences, School of Medicine, Medical Sciences & Nutrition, University of Aberdeen, Health Sciences Building, Foresterhill Road, Aberdeen AB25 2ZD, UK; Health Psychology group, Institute of Applied Health Sciences, School of Medicine, Medical Sciences & Nutrition, University of Aberdeen, Health Sciences Building, Foresterhill Road, Aberdeen AB25 2ZD, UK; Department of Nursing and Midwifery, University of the Highlands and Islands, Centre for Health Science, Old Perth Road, Inverness IV2 3JH, UK; School of Health Sciences, University of Dundee, 11 Airlie Place, Dundee DD1 4HJ, UK; Health Psychology group, Institute of Applied Health Sciences, School of Medicine, Medical Sciences & Nutrition, University of Aberdeen, Health Sciences Building, Foresterhill Road, Aberdeen AB25 2ZD, UK; Psychology, School of Applied Sciences, Edinburgh Napier University, Sighthill Campus Sighthill Court, Edinburgh, EH11 4BN, UK

**Keywords:** Covid-19, adherence, behaviour change, restrictions

## Abstract

It is assumed that increases in Covid-19 cases are caused by people not adhering to advised individual transmission-reducing behaviours. Upon the implementation of restrictions, the hypothesis is that those individuals will change their behaviour. We aimed to retrospectively explore adherence to physical distancing before and after restrictions (e.g., lockdowns) were implemented in a region of Scotland. We assessed adherence, intention, and self-efficacy to physical distancing in a series of cross-sectional telephone surveys of a representative sample of adults in Scotland. We included data from before regional restrictions and after restrictions and examined whether regions with and without restrictions differed in adherence. A total of 1724 Scottish adults (675 men, *M* age = 52.79 years, SD = 17.92) participated (879 (51.0%) pre-restriction, 466 (27.0%) from a restricted region). ANOVA showed that none of the main effects (for region or time) nor the interaction effect were significant. There was a main effect of time on self-efficacy, such that self-efficacy was lower post-restriction measures (*M* = 4.13, SD = 0.81) compared to pre-restriction time (*M* = 4.22, SD = 0.79). There was no evidence that adherence was weaker before restrictions were implemented in regions with higher case rates. Nor was there evidence that imposing restrictions increased adherence. In a future pandemic, it is advisable to assess behaviour and beliefs about Covid-19, risk, and behaviours on an ongoing basis and to use that as indicators of the need for intervention even before cases rates start to go up.

## Teaser

Looking back there was no evidence that adherence was weaker before local restrictions were implemented by government in regions with higher case rates, nor was there evidence that imposing restrictions increased adherence.

**Implications**

**Practice:** Communicating the need for restrictions, increasing targeted behaviours is not easy, and messaging about restrictions should probably be assessed for effectiveness.

**Policy:** In situations where the public is scrutinizing national restrictions, policymakers should consider communicating more clearly on reasons for the restrictions, as looking retrospectively there is little evidence for difference on a behavioural level for behaviour targeted in the restrictions.

**Research:** Future research in acute outbreaks should assess behaviour and beliefs about Covid-19, risk on an ongoing basis, and identify indicators of the need for intervention even before cases rates start to go up.

## Introduction

In response to the Covid-19 pandemic, governments implemented two non-pharmacological measures to reduce coronavirus transmission; “restrictions” such as lockdowns that included curfews, closure of shops, bars, restaurants and sporting events, and stay at home rules, and guidance around individual behaviours such as physical distancing, hand washing, and wearing face covering (e.g., [1–3]). Some of the measures were selectively applied when certain regions had spikes in Covid-19 cases.

Although these two types of measures were implemented to different extents globally, there is a lack of understanding about when to implement these restrictions and the likely influence of restrictions on adherence to transmission-reducing behaviours. Also, there is a lack of studies exploring location specific intervention in such a global effort. Covid-19 case rates have been used to determine guidelines, when and where an increase in cases is noticed, new stricter directives are implemented [2]. It is assumed that increases in Covid-19 cases are caused by people not adhering to advised individual transmission-reducing behaviours. Upon the implementation of restrictions, the expectation is that people will change behaviour and case rates will go down. However, whether behaviour follows this pattern is unclear but can be investigated by comparing adherence to transmission-reducing behaviours of people living in those restricted regions, with the behaviours of people in regions without these restrictions.

Restrictions can do what they are meant to do, local lockdowns, and stay home restrictions, could reduce contact between people and increase physical distancing. However, restrictions could also have possible unintended, undesirable consequences, for example when going out for permitted reasons (e.g., basic necessities, exercise) people might keep less physical distance as they feel the risks are already mitigated. Alternatively, restrictions may have no effect.

Restrictions aimed to curtail Covid, were largely through individual behaviours, such as physical distancing, handwashing, wearing face covering, and latterly vaccination. Government policy was used both to legally enforce as well as guide these behaviours. With regards to physical distancing, at key points in the pandemic there were legally enforced restrictions of people gathering in public places and in their own homes, the guidance on the other behaviours remained the same. When not enforced, guidance about physical distancing remained in place. Hence, the pandemic posed an opportunity for researchers of behavioural medicine to study the impact of restrictions on behaviours to prevent illness.

The present study retrospectively explores data on adherence to transmission-reducing behaviours in participants in the CHARIS project [4] to assess the influence of regional restrictions on adherence to keeping 2 m physical distance. We aimed to explore adherence to physical distancing (self-reported behaviour or intentions) and self-efficacy for adhering to the transmission-reducing behaviours before and after restrictions were implemented and lifted in a region of Scotland and compare it to other regions where no restrictions were implemented. In addition, we assessed other transmission-reducing behaviours that were not directly targeted by the restrictions for potential spill-over effects.

## Methods

### Participants

Participants were included from the CHARIS project, described in detail elsewhere [4]. Briefly, the CHARIS project was a weekly and later fortnightly repeated representative cross-sectional study starting in March and ending in November 2020. All adults aged 16 or older, able to speak English, and currently living in Scotland were eligible to participate. CHARIS was administered by a commercial telephone polling company (Ipsos MORI Scotland).

### Measures

#### Region

Regional restrictions took place between 1 September and 11th (East Renfrewshire, Glasgow, West Dunbartonshire, Renfrewshire, East Dunbartonshire, North, and South Lanarkshire) [5, 6]. We coded all people according to postcode, those in one of the *restricted regions* were coded 1, and those who lived in other regions were coded 0.

#### Time

We included data from before the regional restrictions, *pre-restriction time*: we took data from the weeks between the 30th of July and the 26th of August, coded 0. *Post-restriction time*: we took data from the weeks between 10th September (the last restrictions started one day after this inclusion of participants) and the 7th of October, coded 1.

#### Adherence

People were first asked if they went out of their home in the past week [yes/no], to those who went out we asked about adherence to keeping 2 m distance was assessed by asking: “In the past week, you stayed 2 m (6 feet) away from other people, except those who live in your household,” on a 5-point scale from never, rarely, sometimes, most of the times, to always.

In addition, we assessed handwashing and wearing face covering on the same scale. Handwashing was measured using four items: washing hands as soon as you get home; washing hands using soap and water; washing hands for at least 20 s and washing hands before eating and drinking. Face covering wearing was measured using two items: face covering wearing when in a shop and when travelling on public transport. We calculated two aggregate adherence scores by averaging across the items, higher scores indicated greater adherence.

*Intention and self-efficacy* related to keeping 2 m distance was assessed by asking: “Do you intend to follow all the government instructions…” and “How confident or not are you that you can follow the government instructions…,” “on each of the following. Staying 2 m (6 feet) away from other people, except those who live in your household” on a 5-point scale (1 = never, 5 = always) and on a 4-point scale (1 = not at all confident, 4 = very confident).

### Statistical analyses

In the time pre- and post-restriction 2007, Scottish adults participated, of whom 1724 (86.1%) gave consent to use their postcode information, which could be used to determine their region and could therefore be included in the analyses. We analysed the data using a general linear model (GLM). We conducted a 2 (Region: restricted vs. unrestricted) x 2 (Time: pre- vs. post-restriction) analysis of variance (ANOVA) with region and time as categorical between-subject factors and adherence, self-efficacy, or intention as continuous dependent variables. Significant interaction effects were further analysed with simple comparisons of values 1 SD above and below the mean [7].

## Results

### Participants

A total of 1724 Scottish adults, of whom 675 men, *M* age = 52.79 years, SD = 17.92) participated, 1669 participants (97.2%) were white, and were on average on the 6.13 decile (SD = 2.71) on the Scottish Index of Multiple Deprivation (SIMD) that groups 6,976 postcodes on level of deprivation (1 = most deprived; 10 = least deprived). A total of 879 (51.0%) participants pre- and 845 (49%) post-restriction. In total, 466 (27.0%) from restricted regions (259 (55.6%) pre-restriction, and 207 (44.4%) post-restriction) and 1258 (73.0%) from unrestricted regions (620 (49.3%) pre-restriction and 638 (50.7%) post-restriction).

### Keeping 2 m distance: the effect of time and region on adherence, self-efficacy, and intention

**Figure 1:**
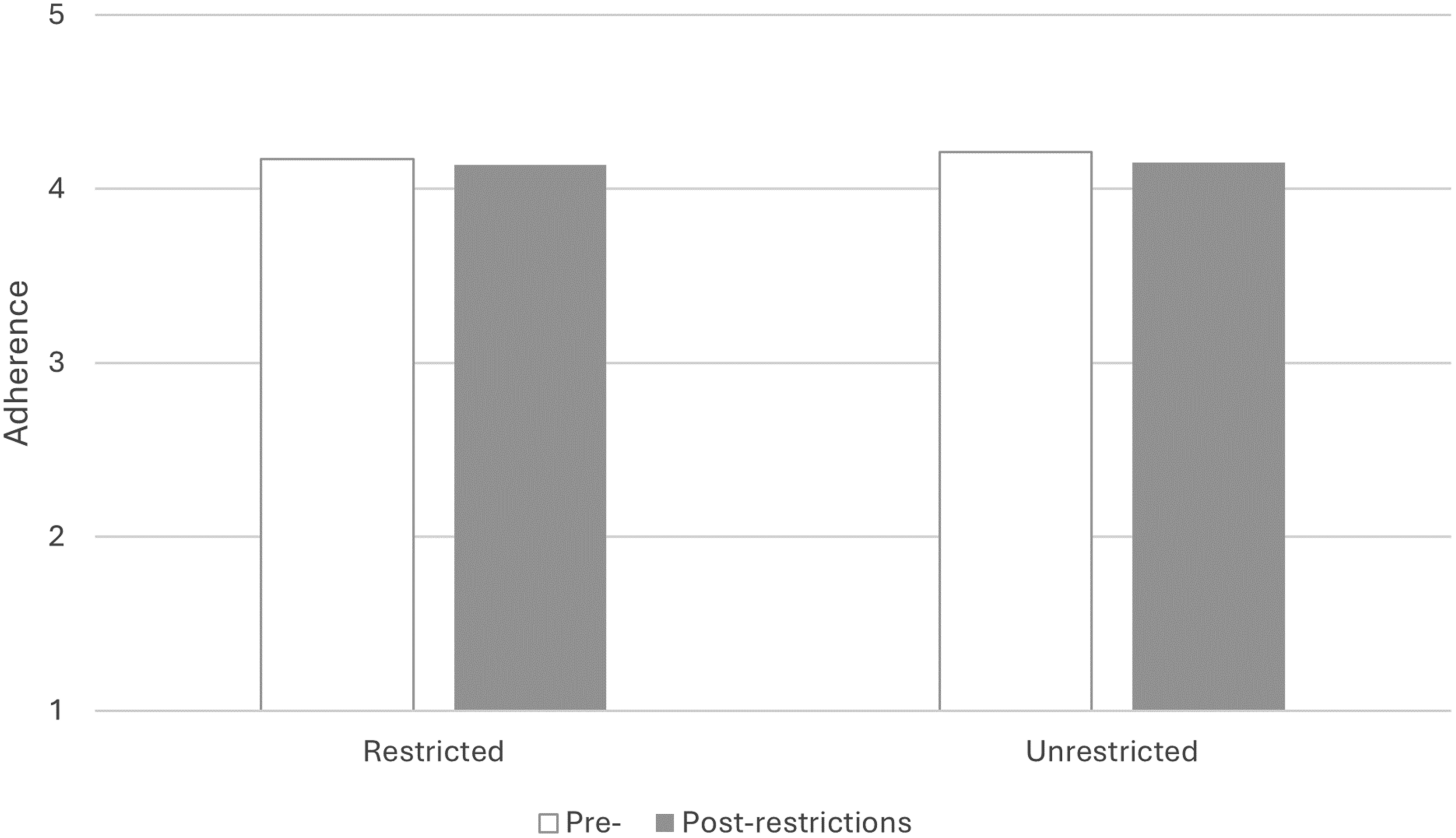
Adherence to keeping 2 m distance for people from restricted and unrestricted regions, and pre- and post-restriction in Scotland

None of the main effects (for region or time) or the interaction effect on adherence were significant, there was no effect of region on adherence to keeping 2 m distance, no effect of time on adherence, and these did not interact such that adherence increased more for people in restricted regions (Fig. 1 and Table 1). Controlling for the sociodemographic variables did not change this pattern of results (data not shown). Age was the only significant covariate. Repeating this analysis on the binary variable for going out, we also did not find any statistically significant main or interaction effects.

**Table 1:** Adherence, self-efficacy, and intention scores (mean and standard deviations unless indicated otherwise) for people from restricted and unrestricted regions, pre- and post-restrictions and outcome for ANOVA

		Time		
		Pre-	Post-restrictions	
Outcome	Region	Mean (SD)	Mean (SD)	Test results
Adherence-physical distancing				Region: *F*(1,1635) = 0.20, *P* = .66, partial-η^2^ = 0.00
	Restricted	4.17 (0.87)	4.14 (0.98)	Time: *F*(1,1635) = 0.63, *P* = .43, partial-η^2^ = 0.00
	Unrestricted	4.21 (0.86)	4.15 (0.90)	Region * Time: *F*(1,1635) = 0.09, *P* = .76, partial-η^2^ = 0.00
Self-efficacy				Region: *F*(1,1711) = 1.29, *P* = .26, partial-η^2^ = 0.00
	Restricted	4.20 (0.80)	4.08 (0.86)	**Time: *F*(1,1711) = 5.01, *P* = .03, partial-η** ^**2**^ **= 0.00**
	Unrestricted	4.22 (0.79)	4.15 (0.79)	Region * Time: *F*(1,1711) = 0.35, *P* = .55, partial-η^2^ = 0.00
Intention				Region: *F*(1,1716) = 1.14, *P* = .29, partial-η^2^ = 0.00
	Restricted	4.24 (0.83)	4.30 (0.88)	Time: *F*(1,1716) = 0.80, *P* = .37, partial-η^2^ = 0.00
	Unrestricted	4.31 (0.79)	4.33 (0.80)	Region * Time: *F*(1,1716) = 0.33, *P* = .57, partial-η^2^ = 0.00
Going out (binary variable)^a^				Region: aOR 1.21 (0.53–2.74)
	Restricted	8 (3.1)	11 (5.3)	Time: aOR 1.08 (0.60–1.96)
	Unrestricted	23 (3.7)	22 (3.4)	Region * Time: aOR 0.53 (0.17–1.59)
Adherence (incl. people who did not go out)				Region: *F*(1,1715) = 0.05, *P* = .83, partial-η2 = 0.00
	Restricted	4.24 (0.92)	4.26 (1.05)	Time: *F*(1,1715) = 0.12, *P* = .73, partial-η2 = 0.00
	Unrestricted	4.29 (0.92)	4.23 (0.96)	Region * Time: *F*(1,1715) = 0.58, *P* = .45, partial-η2 = 0.00
Adherence (binary variable)^a^				Region: aOR 1.18 (0.79–1.78)
	Restricted	208 (83.5)	161 (83.0)	Time: aOR 1.22 (0.89–1.67)
	Unrestricted	504 (85.7)	505 (83.1)	Region * Time: aOR 0.85 (0.47–1.54)
Adherence-handwashing				Region: *F*(1,1643) = 1.13, *P* = .29, partial-η2 = 0.00
	Restricted	4.62 (0.45)	4.57 (0.47)	Time: *F*(1,1643) = 0.15, *P* = .70, partial-η2 = 0.00
	Unrestricted	4.55 (0.54)	4.58 (0.47)	Region * Time: *F*(1,1643) = 2.18, *P* = .14, partial-η2 = 0.00
Adherence-facemask wearing				Region: *F*(1,1542) = 3.36, *P* = .07, partial-η2 = 0.00
	Restricted	4.91 (0.41)	4.83(0.74)	Time: *F*(1,1542) = 0.09, *P* = .77, partial-η2 = 0.00
	Unrestricted	4.89 (0.53)	4.95 (0.34)	Region * Time: *F*(1,1542) = 6.23, *P* = .01, partial-η2 = 0.00

Second part of the table shows the scores for the sensitivity analyses.

aOR = adjusted odds ratio (in brackets 95% confidence intervals).

^a^Reported *N* (%) adherent (most or all of the time) and multiple regression analysis.

There was a main effect of time on self-efficacy, such that self-efficacy was lower post-restriction measures (*M* = 4.13, SD = 0.81) compared to pre-restriction time (*M* = 4.22, SD = 0.79) independent of region. The main effect for the region or the interaction did not significantly influence self-efficacy. None of the main effects or interaction were significantly associated with intention to keep 2 m distance.

### Sensitivity analyses

Including people who did not go out in the previous week, as being very adherent to keeping distance, did not change the pattern of results. None of the main effects (for region or time) or the interaction effect reached significance (Table 1). Looking at the binary variable for adherence (adherent most of time and always versus less or non-adherent) did not change the pattern of results.

### Handwashing and face covering wearing

In addition, there were no effects (main or interaction) of adherence to handwashing. There was no effect of region on handwashing adherence, no effect of time on adherence, and these did not interact such that adherence increased more for people in restricted regions. Adherence to wearing face covering did show an interaction effect. Simple slope analyses showed that people in the non-restricted regions were more adherend to wearing face covering post-restriction compared to pre-restriction, *F*(1,1542) = 4.27, *P* = .04, partial-η^2^ = 0.00. The two regions only differed in adherence post-restriction, *F*(1,1542) = 8.68, *P* < .01, partial-η^2^ = 0.00.

## Discussion

The CHARIS project did not find that regions with tighter restrictions were less adherent before the restrictions compared to other regions, nor were they more adherent after restrictions compared to their pre-restriction behaviours and compared to other regions. These findings remained whether looking at adherence to physical distancing, or the stricter staying inside your home. In addition, there were no effects on intentions to physical distance or self-efficacy in physical distancing. We only found that people decreased in self-efficacy over time independent of the region they were living in; in other words, people became less confident in their ability to keep physical distance from other people over time.

Assessing whether the restrictions also affected other transmission-reducing behaviours, showed that they did not affect handwashing, but did affect wearing facemasks. Face covering. Face covering wearing increased in regions where there were no restrictions. One might expect spill-over effects, when adhering more to physical distancing people also increase adherence to other transmission-reducing behaviours, or negative effects by adhering more strictly to physical distancing the perceived need to adhere to other transmission-reducing behaviour is viewed as unnecessary. Neither is what we found, instead people in non-restricted areas seemed to be changing their behaviour after the restrictions were implemented and imposed on other regions. This could reflect people taking precautions to prevent more restrictions in their area or increased risk perception as communicated by these restrictions.

Studies show that some restrictions and guidelines on a national level did demonstrably affect behaviours. For example, when face covering became mandated, there was a significant rise in adherence to wearing face covering guidelines [8]. Another study using GPS-derived data showed that people changed their movements outside of their home in response to national restrictions [9]. Studies also showed that adherence to transmission-reducing behaviour was not perfect and varied depending on the specific behaviour [10].

The Capability, Opportunity, Motivational-Behaviour (COM-B) model of behaviour change suggests that adherence to transmission-reducing behaviours will be influenced by individual capability, opportunity, and motivation [11]. Applied to understanding the impact of restrictions on transmission-reducing behaviours, it is conceivable that restrictions may increase “motivational” factors that influence these behaviours by for instance, re-enforcing beliefs about the severity of the illness Covid-19 and vulnerability to the disease and hence, and in this scenario, transmission-reducing behaviours would increase in regions with restrictions but not necessarily in regions without restrictions. However, the restrictions could also remove people’s “opportunity” to keep 2 m distance, thereby reducing people’s personal mastery experiences and maybe even reducing “capacity” to engage in physical distancing, because the confidence to do that decreases. The current findings do show that self-efficacy decreased over time, but this was not region specific.

Of course, there are other factors besides self-efficacy that could affect behaviour change, above we already mentioned beliefs about the risk as operationalized in for example protection motivation theory [12]. Indeed, we have shown that these factors and other social cognitive factors are associated with adherence in this population [8]. In addition, we think many environmental and social factors, including social support affect adherence [i.e., 13]. However, we think these potentially are a more likely a result of physical distancing behaviour (not a predictor) and indirectly influenced by the policy changes, as opposed to directly affected by the policy changes.

Strengths of this study are that we were already assessing adherence to transmission-reducing behaviour before the regional restriction were introduced, giving us the opportunity to compare *within* the restricted region before and after the restrictions and with regions without restrictions. Participants were recruited using random selection procedures resulting in more representative samples than via opportunistic or online recruitment. A limitation of this study is that it is a repeated cross-sectional survey, we did not assess behaviour of the same people later in time. Our methods are indicative of needing to rapidly conduct research during a pandemic, and reflects an approach widely adopted in the field to be able to start assessing behaviour as soon after the outbreak as possible [14]. In a systematic review, we found this study design was prevalent and posit that follow-up in the same people would improve validity and behaviour change [14]. However, restrictions that were implemented aim to change behaviours of the whole community, and the effects of such restrictions should be visible in a community sample such as ours.

In addition, all limitations related to self-report measures are applicable to the current study. Some of the measures just included one item, because of limited resources, and to minimize respondent burden. Of course, it would improve study validity to have access to objective data. We did not plan to include observational data, and we cannot retrospectively add any measure as our study includes the entirety of Scotland, which means even if we find suitable observational data matching to our self-report would be incredibly time consuming. Nevertheless, in some countries anonymous aggregated mobile phone GPS data, or other wireless proximity data, was used to assess adherence to restrictions [9]. Other objective physical distancing measures that could be considered in future research are location specific, room-entry data for hospitals, Wi-Fi connected device to calculate a population density in particular areas. Or proxies heavily affected by context, such as number of police citations, or complaints on social media. These technologies currently have extensive limitations, associated with costs, privacy, and accuracy, and are not fit to be widely and timely used for physical distancing observation [15]. We know that self-report is likely to over-estimate adherence to most protective behaviours [16], however even these researchers concluded self-report is a useful proxy for behaviour and there are valid reasons for choosing to use it (i.e., speed, costs, ease). When new outbreaks occur, researcher could consider planning to combine self-reported data, with one or more of these more objective measures.

The current study applies a behavioural science perspective to one particular policy and assesses how people responded in the affected and unaffected regions. Future work could collect, categorize, and analyse government policy implementation. Additional attention could be given to jurisdiction, level of authority, variance among legal elements, consequences for non-compliance, or enforcement provisions associated with government policies.

The guidelines emphasized limiting contact with people from other households, especially indoors, and restricted travelling to other regions; meeting outdoors was permitted as long as the other guidelines such as physical distancing were upheld. The increased case rates were attributed to meeting and interacting indoors [14], which suggest lack of physical distancing. However, the increase in cases in the regions that got restrictions might have been caused by other behaviours (we did not assess), or by a general increase in prevalence of Covid-19 that makes any contact with people higher risk for Covid-19 transmission, or because people in that region tested more and thus more Covid-19 cases were found. Our findings have shown that a common-sense explanation of increased cases might not fit, and the situation is more complex. Therefore it would be advisable in a future pandemic to assess behaviour and beliefs about Covid-19, risk, and behaviours on an ongoing basis and to use those as indicators of the need for intervention even before case rates start to go up [13].

## Welfare of Animals

This article does not contain any studies with animals performed by any of the authors.

## Transparency Statements

1. This study was not formally registered. However, the study was conducted as part of the CHARIS (Covid-19 Health and Adherence In Scotland) project and the published protocol describes the samples, main measures and proposed analyses (den Daas *et al*. 2021).

2. The analysis plan was not formally pre-registered.

3. De-identified data from this study are not available in an a public archive. De-identified data from this study will be made available (as allowable according to institutional IRB standards) by emailing the corresponding author.

4. Analytic code used to conduct the analyses presented in this study are not available in a public archive. They may be available by emailing the corresponding author.

5. Some of the materials used to conduct the a study are available in a public archive: https://www.ncbi.nlm.nih.gov/pmc/articles/PMC7896376/bin/bmjopen-2020-044135supp003.pdf).
